# Enhancing the design, conduct and evaluation of public health emergency preparedness exercises: a rapid review

**DOI:** 10.1186/s12889-025-23270-6

**Published:** 2025-07-03

**Authors:** Andrea Chambers, Isra Khan, Ruth Repchuck, Sarah Muir, Heather Hanson, Yasmin Khan

**Affiliations:** 1 Public Health Ontario, Toronto, ON Canada; 2 Division of Emergency Medicine, Department of Medicine and Clinical Public Health Division, Dalla Lana School of Public Health, University of Toronto, Toronto, ON Canada

**Keywords:** Emergency preparedness, Emergency management, Public health, Resilience, Exercise, Simulation, Tabletop, Drill

## Abstract

**Background:**

Emergency preparedness exercises are essential in building a resilient public health system that can support increasingly frequent and complex public health risks. This rapid review describes evidence-informed practices or principles that can enhance all-hazards emergency preparedness exercises, with a specific focus on the public health agency context.

**Methods:**

Four databases were systematically searched, including MEDLINE, Embase, Global Health, and CINAHL (January 2013-June 2024) and complemented with a grey literature search. Studies were included if published in English, from a member country of the Organisation for Economic Co-operation and Development, relevant to the public health agency context, and reported outcomes that aligned with our primary research question. Included articles were assessed for quality based on study design. The analysis involved a descriptive summary and thematic analysis.

**Results:**

The review identified fifteen studies that reported insights on optimizing exercise design and delivery, with most studies reporting on tabletop exercise evaluation results. We identified ten sub-themes on how to strengthen exercises with a focus on how scenarios are developed, how participants are selected and organized during an exercise, thoughtful selection and training of exercise facilitators, selecting evaluation methods that closely align with the purpose of the exercise, and the importance of embedding activities that will encourage pathways to improvement.

**Conclusions:**

Findings from this review can be utilized in practice to enhance the design of emergency preparedness exercises. There are several gaps that should inform future work, including the need for additional studies focused on exercises conducted within the public health agency context, as well as more rigorous research to strengthen knowledge regarding evidence-informed practices in this area. There is a need for more guidance on triggers for conducting exercises, additional research on innovative technologies and approaches to enhance participant engagement, and guidance on incorporating evidence-based frameworks and indicators to improve exercise design and evaluation.

**Supplementary Information:**

The online version contains supplementary material available at 10.1186/s12889-025-23270-6.

## Background

As public health systems emerge from response to the Coronavirus Disease 2019 (COVID-19) pandemic, new risks continue to require preparedness and response, from highly pathogenic avian influenza to wildfires [1, 2]. In this current context of emerging and re-emerging infectious diseases and serious health impacts from climate change, public health risks are increasingly frequent and complex [3, 4]. Building resilience to support corresponding frequent and complex emergency management activities is important for the public health system. An essential element in a framework to promote resilience in the public health system for all-hazards emergencies is Practice and Experience [5]. This element describes how through experience, public health agency roles and responsibilities can be clarified, relationships can be developed, and planning processes can be refined and enhanced [5]. Experience may be gained through responses to public health incidents and emergencies or, in the absence of active responses, through simulated incidents and/or emergencies.

Simulated scenarios or exercises are a well-established component of health system emergency management programs [6–9]. Exercises can be designed to test existing plans or policies, specific skills or tasks, specific functional areas, or an organization’s capability or structure and can take different forms including those that are more discussion-based (e.g., tabletop exercises) or operations-based (e.g., drills, functional exercises, full-scale exercises) [10, 11]. Through exercises, participants can be additionally challenged with the evolution and increasing complexity of a scenario; for example, factors that influence decision-making in the allocation of limited resources.

A number of individual-level benefits of exercises have been described in the literature including improvements in knowledge, competencies, and confidence in emergency response activities, policies, and procedures among participants [11–13]. At an organizational level, exercises have been found to help in identifying gaps and opportunities to improve emergency plans, protocols, and procedures [11, 14–17]. Simulated scenarios or exercises can support feedback and co-evolution amongst individuals and organizations supporting adaptation and improvements overtime with the goal to strengthen public health system capacity and resilience [5]. The impact of emergency preparedness exercises on performance in real-life emergencies is an underdeveloped area of research, primarily supported by a few studies conducted in healthcare settings or with a focus on first responders [11, 14–18].

While summarizing the benefits of exercises is important to reinforce their value as a preparedness activity, recent reviews have not synthesized information on optimizing exercise design and execution to identify core principles and practices which could be useful to enhance exercise outcomes [11, 14]. Since development and implementation of exercises is resource-intensive, it is vital to ensure exercise development is informed by evidence to ensure desired outcomes are achieved.

To date, reviews on this topic have primarily focused on exercises conducted within healthcare settings, with limited attention to the public health agency context [11, 14]. This is an important gap considering the essential role public health agencies play in health system emergency management, and the importance of exercises in strengthening preparedness and resilience for emerging or re-emerging public health risks. Thus, the aim of this rapid review was to identify what evidence-informed practices or principles can enhance all-hazards emergency preparedness exercises. The specific question of interest was: What are evidence-informed practices or approaches that can enhance the design, delivery and evaluation of all-hazards emergency preparedness exercises in the public health agency context?

## Methods

### Design

A rapid review method was selected to collect and synthesize literature in the current context of frequent and dynamic public health risks. The Cochrane Rapid Review Methods Group has defined rapid reviews as follows: “A rapid review is a form of knowledge synthesis that accelerates the process of conducting a traditional systematic review through streamlining or omitting various methods to produce evidence for stakeholders in a resource-efficient manner [19].” This review was conducted with a local or regional public health agency in mind recognizing a decrease in capacity and resources dedicated to this activity during the COVID-19 pandemic and a renewed interest in, and recognition of, the importance of emergency preparedness exercises. Due to an immediate need to consider strategies to strengthen preparedness for highly pathogenic avian influenza, amongst other risks, a rapid review methodology was selected to be able to gather timely and actionable insights on evidence-informed practices for emergency preparedness exercises. Specific details on how traditional systematic review methods were adapted to align with a rapid review approach are described below [19]. While the Preferred Reporting Items for Systematic Reviews and Meta-Analyses (PRISMA) Statement is not yet available for rapid reviews, relevant items were reviewed to guide the reporting of this work (See additional file 1) [20].

### Databases and search strategy

Four databases, including MEDLINE, Embase, Global Health, and CINAHL, were systematically searched in June 2024 using a librarian-assisted search strategy. Search terms included exercise terms (e.g., tabletop, simulation, operations-based exercise); hazard-specific terms; emergency-related terms (e.g., disaster, pandemic, outbreak); public health-related terms; and key terms related to methods, including evaluation approaches (e.g., lessons learned, after-action review). The search strategy was restricted to studies conducted by member countries of the Organisation for Economic Co-Operation and Development (OECD) to ensure insights gathered reflected contexts that are more comparable to Ontario, Canada in terms of economic development, governance structures and public health system capacities [21]. The search was limited to articles published in English based on the language capacity of the review team and the limited time and resources available for translation of studies [22]. The search was also limited to articles published since 2013 to yield a feasible volume of search results. A grey literature search was also conducted to identify any additional primary studies for this review. See additional file 2 for the full indexed literature search strategies. Studies included in existing systematic or scoping reviews on emergency preparedness exercises were reviewed to assess relevance to this study’s review question.

### Inclusion and exclusion criteria

To be eligible for inclusion, articles had to focus on public health emergency preparedness exercises and report results of a research study or evaluation. The included studies within secondary research papers on the topic of emergency preparedness exercises that had a different focus in their analysis (e.g., summarizing the outcomes of exercises) were closely examined for relevance for this review. Relevant studies were not excluded if they were published before 2013. Studies were excluded if they only reported results on the outcomes of the exercise (e.g., whether objectives were met, what practice gaps were identified) without any linkage to, or reflection on, specific practices or principles that could enhance exercise effectiveness.

In order to identify key findings that would be relevant to the public health agency context, articles describing exercises conducted exclusively in healthcare facilities to improve healthcare delivery or response without a public health practice component were excluded. Articles were also excluded if they were exclusively conducted within an academic or training or educational context. Exercises that had both a healthcare and public health practice component were, however, included.

Studies were imported into Covidence software [23]. Two reviewers were involved in the title and abstract screening process using the inclusion and exclusion criteria. In line with rapid review approaches and to ensure consistency and reliability in study selection, 20% of the search results were screened by both reviewers in order to achieve agreement on the application of the screening criteria. Once agreement was achieved, reviewers screened the remaining results independently [19]. The same process was used to complete the full-text review to assess eligibility. Reasons for exclusion were recorded in Covidence. Group discussion was used to reach agreement on the final set of articles.

### Data extraction and analysis

For all articles included in this review, the aim, type of study, data sources and analysis approach were described and relevant details on exercise design and outcomes were extracted. Relevant outcomes in scope for the analysis included any results from the study that identified exercise design best practices or lessons learned relevant to foundational planning, exercise design, facilitation of exercises, and evaluation and improvement. The analysis focused exclusively on qualitative data extracted as key insights from qualitative, evaluation and mixed-methods studies. While some included studies also contained quantitative data, these components did not relate to our research question and were, therefore, not considered in the synthesis. Data extraction was completed by a single reviewer and validated by a second reviewer [19]. As the review did not place any restrictions on study design, the data sources and analysis approaches informing the outcomes of interest were closely examined to select appropriate critical appraisal tools. To ensure a relevant and consistent assessment of included studies, a well-established critical appraisal tool for qualitative studies from the Critical Appraisal Skills Programme (CASP) was selected as a foundation [24]. This appraisal tool has been adapted successfully in other reviews that have synthesized qualitative insights extracted from various study designs [25, 26]. The criteria were reviewed and adapted to enhance applicability to mixed method and evaluation studies. Additionally, as our review included secondary research, a separate critical appraisal tool from CASP designed for systematic reviews was used [27]. This ensured that the unique methodological and reporting considerations for evidence synthesis studies were appropriately assessed (see additional file 3 for an overview of the quality criteria used and results). Articles were assessed by a single reviewer to identify common methodological issues and opportunities to strengthen the reporting and conduct of studies in this area.

An inductive thematic synthesis was used to identify subtopics and themes relating to key insights on exercise design [28]. This involved inductive coding of the findings from the primary studies, organization of these codes into descriptive themes and then further development of analytical themes [28]. Initial themes were generated by one reviewer, followed by an iterative process of analysis with three other reviewers.

## Results

### Search results

**Fig. 1:**
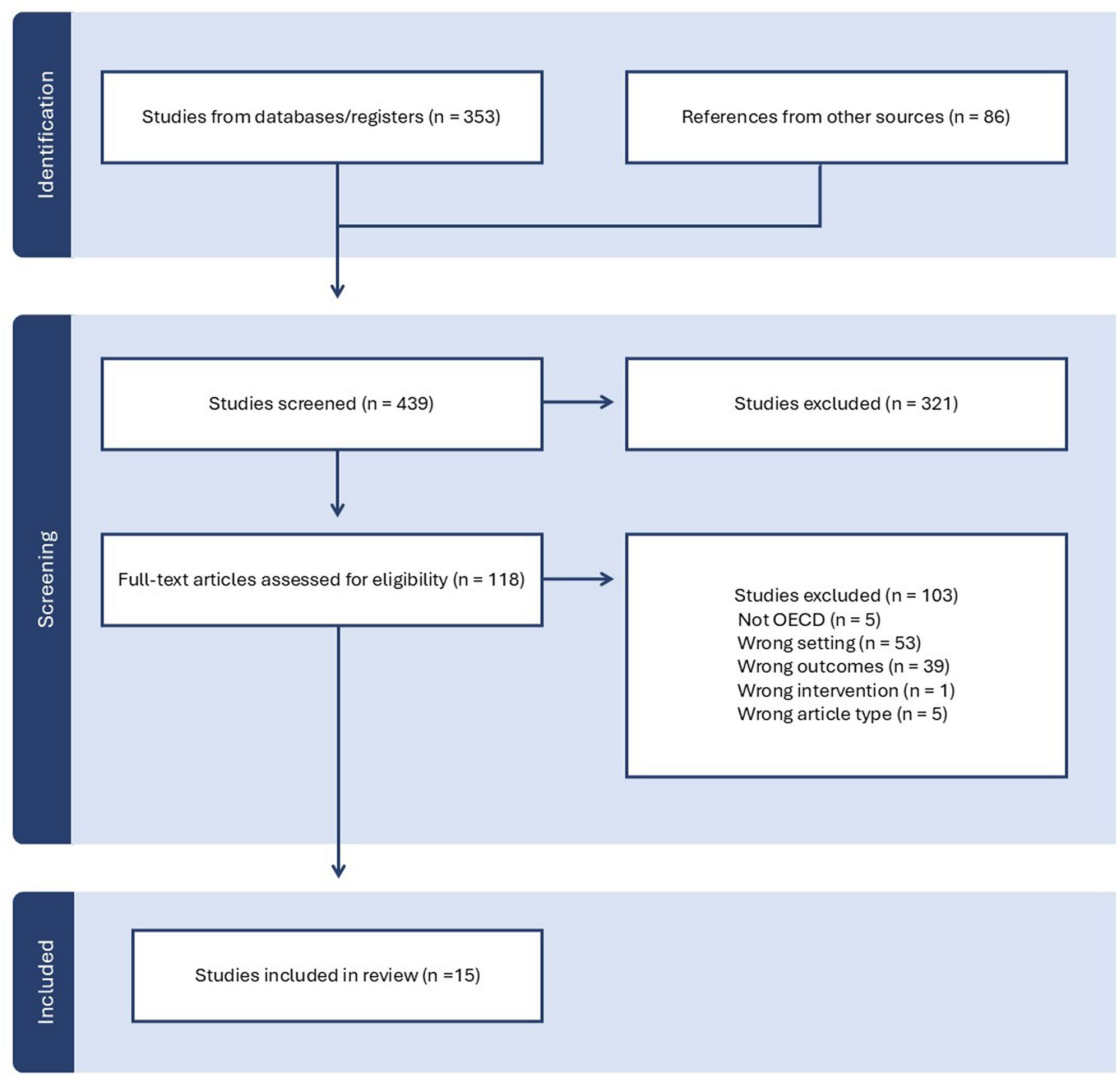
Article screening flowchart based on the PRISMA guidelines

The indexed literature search returned 353 results. An additional 86 studies from a scoping review published in 2017 by Skryabina et al. were reviewed for relevance to the public health agency context [11]. After title and abstract screening, 118 were included in the full-text review and 15 studies met the inclusion criteria and were part of the final set of included articles (see Fig. 1).

### Description of included studies

Table 1 provides a description of the included studies including the aim, study type, data sources and analysis approach.

**Table 1:** Description of the study aim, type of study, data sources and analysis approach for included studies (*n* = 15)

Author [Year]	Aim	Type of study	Data sources	Description of analysis approach supporting relevant key findings for this review
Dausey (2007) [39]	To identify lessons learned about developing and conducting 31 tabletop exercises in collaboration between RAND researchers and state and local health departments in the United States (US).	Descriptive (qualitative)	Document review (participant self-evaluations, after-action reports, evaluation forms, debriefs)	Identification of themes across data sources and reflections from the authorship team presented as “lessons learned”
Freimuth (2008) [29]	To describe the design, implementation and evaluation of an exercise designed to exercise risk communication competencies.	Descriptive (single exercise evaluation)	Participant survey and author (exercise developers) reflections	Reflections from the authorship team (exercise developers)
Ghiga (2021) [40]	To describe and discuss the utility of the application of a gamified simulation exercise aimed at strengthening national pandemic vaccine preparedness and readiness, and to present findings from the evaluation.	Descriptive (exercise evaluation tested with multiple groups)	Participant feedback during a debrief and post-exercise questionnaire (*n* = 79)	Summary of participant feedback
High (2010) [30]	To describe the design, implementation and evaluation of an exercise designed to test preparedness for chemical disasters.	Descriptive (evaluation of four exercises)	Documentation (attendance rosters), post-exercise surveys, post-exercise debriefs	Author (exercise developers) recommendations based on the evaluation results
Macario (2007) [31]	To describe an approach to emergency response training that incorporates a distance-learning-by-satellite component with locally managed activities and to describe lessons learned from applying this approach to an avian influenza pandemic scenario.	Descriptive (single exercise evaluation)	Participant survey (*n* = 164), a facilitator survey (*n* = 12) and a follow-up teleconference with local health departments	Reflections from the authorship team presented as “lessons learned” based on evaluation results and experience
Macario (2009) [32]	To describe a broadcast/training offering and associated tabletop exercise focused on pandemic influenza and to share lessons learned from the process.	Descriptive (single exercise evaluation)	Evaluation forms completed by participants (*n* = 225) and feedback from training facilitators	Overall reflections from the authorship team and summary of participant feedback
Manageiro (2023) [33]	To describe the design and outcomes of a tabletop exercise that practiced One Health capacity and interoperability across public health, animal health, and food safety sectors to improve One Health preparedness for future disease outbreaks.	Descriptive (single exercise evaluation)	Post-exercise evaluation survey and reflections from the authors (exercise developers)	Summary of key themes from participant feedback
Morris (2012) [34]	To describe the development, design and outcomes of a tabletop exercise focused on an unusual foodborne outbreak pathogen.	Descriptive (single exercise evaluation)	Evaluator (*n* = 4/6), participant surveys (*n* = 22), exercise developer reflections	Author (exercise developers) reflections on limitations
Obaid (2017) [41]	To describe the design, implementation and outcomes of a series of discussion based and functional exercises (scenarios based on infectious diseases requiring medical care and public health investigation) designed to test decision-making around incident command systems and activities among rural emergency response partners.	Descriptive (evaluation of six exercises)	Participant feedback forms, exercise debriefs and reflections from the authors (exercise developers)	Reflections from the authorship team
Sandrom (2014) [35]	To describe the development and evaluation of a generic tool (exercise cards) for tabletop exercises focused on an intentional or unintentional release of chemical, biological, radiological and nuclear agents.	Descriptive (iterative testing and evaluation of an exercise with three groups)	Observations of the various applications of the tool with different groups/settings	Reflections from the authorship team (exercise developers)
Sarby (2005) [36]	To describe the process used to design, execute, and evaluate a tabletop exercise simulating a response to severe acute respiratory syndrome (SARS) and to report the evaluation results.	Descriptive (single exercise evaluation)	Participant survey (open-ended survey questions) (*n* = 44/49)	Qualitative responses were content analyzed to provide themes related to continuous improvement
Savoia (2009) [37]	To assess the usefulness of combining educational sessions with a tabletop exercise as educational tools in legal preparedness, to assess the impact of the exercise on the participants’ level of confidence about the legal preparedness of a public health system, and to identify legal issue areas in need of further improvement.	Descriptive (single exercise evaluation)	Participant pre-post surveys	Reflections from the authorship team based on the survey results.
Savoia (2014) [42]	To develop a conceptual framework that describes the essential elements necessary to consider when applying performance measurement science to public health emergency exercises.	Descriptive (qualitative)	Document review (from 70 + exercises) and expert consultation (*n* = 61)	Mixed methods: Nominal group technique consultation methods and document review– grounded theory guided development of framework
Skryabina (2018) [43]	As part of a larger scoping study, the aim was to present analysis of evaluation methods used for different types of health emergency preparedness exercises, both operations-based and discussion-based and to provide further evidence on the benefits of specific evaluation methods depending on the purpose of evaluation and exercise content.	Scoping review	Extracted information from 64 studies	Observations of trends and gaps in the conduct of exercise evaluations
So (2019) [38]	To describe the development and outcomes of a virtual tabletop exercise focused on pediatric emergency preparedness and identify exercise strengths and weakness.	Descriptive (single exercise evaluation)	Mixed method pre-post survey with follow-up surveys at 1 and 6 months following the exercise, observation forms completed by independent evaluators, debrief notes, and notes from an after-action review	Qualitative analysis of open-ended feedback from surveys and observational data.

The majority of articles described the design and conduct of a single emergency preparedness exercise and reported the results of the evaluation component (*n* = 10/15) [29–38]. Three additional articles described outcomes from reviewing or conducting multiple exercises [39–41]. Two of the remaining articles were related to evaluation, including a scoping review that analyzed evaluation methods used for different types of health emergency preparedness exercises [42] and an article that presented a comprehensive conceptual framework to guide the evaluation of emergency preparedness exercises [43].

### Quality appraisal

All included articles were critically appraised. Six of the 15 studies provided sufficient information to meet the criteria for over half of the items in the CASP tools (see Additional file 3 for results) [29, 36–38, 42, 43]. Two studies provided adequate information for all critically appraised items [38, 43]. CASP items that were poorly reported across studies included transparency in the role of the researcher; clarity of methods for data collection; clarity of research, evaluation questions or objectives; and clarity of the data analysis process.

### Exercise characteristics

Table 2 provides a summary of descriptive characteristics of the exercises detailed in the included studies, where available and relevant based on the type of study. The majority of the articles reported on the use of tabletop exercises [30, 34–36, 38–40], four of which also incorporated pre-exercise training [31–33, 37]. Two articles described operations-based exercises including a drill to test risk communication capacities and a series of functional exercises to test Incident Command Systems [29, 41]. Included studies focused on scenarios involving infectious hazards (i.e., influenza, avian influenza, Severe Acute Respiratory Syndrome, smallpox) [29, 31–34, 36–38, 40], (i.e., chemical, biological, radiological, and nuclear exposures) [30, 35], or focused on exercises addressing multiple types of hazards [39].

**Table 2:** Descriptive characteristics of exercises in the included studies (*n* = 15)

Study	Exercise type	Scenario type	Hazard(s)	Level of participating organizations
Dausey (2007)	Tabletop	All hazards	Pandemic influenza, smallpox, plague, anthrax, botulism	Local and state
Freimuth (2008)	Drill	Infectious	Influenza	Local
Ghiga (2021)	Tabletop	Infectious	Influenza	National
High (2010)	Tabletop	Non-infectious	Bioterrorism– chemical exposure	Local, state, national
Macario (2006)	Training + tabletop	Infectious	Influenza	National and state
Macario (2009)	Training + tabletop	Infectious	Influenza A/H5N1	State
Manageiro (2023)	Training + tabletop	Infectious	*Salmonella* Typhimurium outbreak	National, central and local levels
Morris (2012)	Tabletop	Infectious	Foodborne outbreak	State
Obaid (2017)	Multiple functional exercises	Infectious	NS	Local (rural)
Sandrom (2014)	Tabletop	Non-infectious	Chemical, biological, radiological or nuclear materials	National and international
Sarby (2005)	Tabletop	Infectious	SARS	State
Savoia (2009)	Training + tabletop	Infectious	Influenza (Avian)	Local, regional, state
Savoia (2014)	N/A	N/A	N/A	N/A
Skryabina (2018)	Multiple exercise types	All hazards	N/A	Local, regional, national
So (2019)	Tabletop	Infectious	Smallpox	National

N/A, not applicable; NS, not specified

### Summary of findings by descriptive category

The synthesis and analysis of the findings are presented below. The subtopics and themes relating to key insights on how to optimize exercises were found to relate to four broad categories including exercise design, participant selection, exercise facilitation and discussion, and exercise evaluation and improvement.

#### Exercise design

##### Establishing a primary aim for the exercise

Design considerations were emphasized in the included studies. Exercise design needs to be informed by a primary exercise aim. It is essential that the primary aim is identified early as this helps direct all design choices including the type of exercise, the design of the scenario, discussion questions or tasks, level of facilitator involvement, and evaluation methods [35, 39, 42]. As exercise design is dependent on this primary aim, it is important to limit the number of aims to avoid the risk of conflicting objectives [35]. Most articles described exercises that have multiple aims, (e.g., training and response plan development/improvement or training and performance assessment) despite this recommendation to focus on a primary aim for the exercise [29, 30, 35, 37, 40]. A few articles focused on exercises designed for training purposes [31, 36, 38, 40] and others focused on exercises designed to improve plans or strengthen response capacities [32–34].

##### Directing exercise content

Based on the experience and outcomes of conducting over 30 exercises, one article recommended that exercises should be designed around priority issue areas (e.g., clarity of roles and responsibilities) identified in past exercises rather than scenarios [39]. Additional success was reported with the use of other types of inputs to direct exercise content including the use of core competencies (e.g., competencies for legal preparedness and more general areas of understanding roles and responsibilities, chain of command, relevant response plans, communication roles in an emergency response) [36, 42]; pre-set objectives (e.g., the need to illustrate teamwork and coordination); and literature reviews on the hazard [30, 36, 37].

##### Embedding educational components

The value of integrating educational components into an exercise to enhance learning outcomes and better support participants in identifying opportunities to improve policies and procedures was also highlighted by multiple articles [31, 32, 37, 40]. Educational supports were used to prepare participants for the exercise such as educational sessions delivered before the exercise [31, 32, 36, 37]. There were also examples of educational materials being delivered during the exercise including supplemental reference materials or videos covering information on the hazard, roles and responsibilities, emergency response plans, in addition to general advice [31, 36, 38].

##### Developing realistic scenarios

There were a number of studies that focused on the importance of crafting detailed and plausible scenarios with a high degree of realism [35, 36, 39, 40, 42]. This included suggestions to tailor exercises to national, regional, or local contexts [33] and even incorporate actual names of individuals and organizations in scenarios [36]. Psychological fidelity refers to the degree to which the scenario prompts cognitive, behavioural and affective responses that would be similar to responses in an actual event [44]. Examples of strategies include mirroring a realistic time frame, incorporating ambiguity with respect to presentation of illness, participants becoming infected, using actual names of individuals and organizations, incorporating contextual factors such as resource availability, having inputs simulate actual response tasks (e.g., risk communication tasks), and incorporating details from real events [21, 30].

Conversely, using fictitious countries or regions that are generalizable can be valuable when creating exercises that can be implemented by many different groups or regions [40]. While these types of packaged exercises can be useful in expanding the number of groups that benefit, this may be at odds with insights highlighted above that emphasize the importance of realism. Expanding the reach of prepackaged exercises may come at the expense of realism and may require adaptation, especially if the exercise aim is to assess performance [43].

#### Selecting and engaging participants

##### Supporting collaboration

Several articles highlighted the strategic value of bringing in a mix of agencies and roles to participate in the exercise. This approach can increase the relevance of the exercise [36], strengthen collaborative relationships [33, 34, 38, 39], test collaboration and coordination [29, 41], help understand the unique contributions of different organizations (including their roles, responsibilities, and capabilities) [30, 33, 34, 38], support more accurate decision-making processes within exercises [42], and ensure multiple points of view are considered [33]. When strengthening relationships is an objective, it is recommended that exercises be organized to support both lateral and vertical discussions among participants [30]. Lateral discussions could involve bringing together participants with similar functions from the same or different organizations. Vertical discussions could involve bringing together various levels of roles within or across organizations.

With respect to team composition, one article asserted that forming teams with a mix of expertise supported teamwork, critical thinking and knowledge exchange [40]. Another article discussed the role of power dynamics between individuals and groups and how some participants may feel less comfortable making mistakes depending on who they are seated with during an exercise [39]. Authors recommended convening participants with similar levels of responsibility into small groups and then have a section in the exercise agenda where groups can share what they discussed and learned [39]. However, it has also been noted that separation into groups by role can impact the ability of participants to address specific types of discussion questions [30]. These perspectives underscore the importance of aligning exercise facilitation and structures with exercise objectives.

##### Reviewing participant representation

Some studies provided strategies to help critically examine participant selection. Examples included reviewing exercise plans with the lens of what types of participants are needed to support discussions during the exercise [36, 40], matching the nature of the discussions and decisions involved in the exercise with the nature of the participants’ roles [30], and describing the rationale for including different professional roles and organizations [32]. This does not necessarily mean increasing the overall number of participants but aiming for thoughtful representation to ensure multiple points of view are considered [33]. Gaps in participant representation were described as impacting the accuracy of decision-making processes during the exercise and inaccurate assumptions about roles and capabilities [42].

#### Exercise facilitation and discussion

##### The role of exercise facilitators

Included studies highlighted the importance of facilitator expertise (e.g., skills in facilitating small group discussion, content knowledge in the area and context) and investing time and resources to train facilitators well in advance of the exercise [31, 32, 35, 36]. Facilitators are essential in encouraging participants to go beyond general answers on complex matters and ensuring exercises are delivered as planned [32, 35].

Among included articles, the level of involvement of facilitators to support the exercise discussions or tasks varied. Some articles described exercises where the facilitators were less involved in small group discussions but played a more involved role in larger group discussions or debriefs [29, 34, 36, 38], while others detailed exercises where the facilitators had more direct involvement in supporting small group discussions, providing guidance and prompts during the process [30–32, 35, 37]. Decisions about the role of the facilitator and level of involvement can be informed by the purpose of the exercise and the complexity of tasks [39]. For example, exercises that have been developed with gamification elements (i.e., use of a game board, procedures, game cards) may benefit from direct facilitator involvement to bring clarity to the process [35, 40]. More limited facilitator involvement may be beneficial when the purpose is to assess performance independent of external support [29].

##### Focusing exercise discussions

A key point highlighted in the literature for discussion-based exercises, is to develop more specific discussion questions that will guide targeted and time-bound decision-making (e.g., should schools be closed at this point?) [29, 30, 40]. It is also important for these questions and decision points to align with participants’ roles and responsibilities. One article found that a key strength of their exercise was how it incrementally brought participants through various stages or “missions” that together helped participants review sections of their response plans [40]. This type of progression can be helpful when participants have less developed plans or where there is a need to identify opportunities to improve existing plans [40]. The importance of allowing adequate time for in-depth discussions or networking was discussed in two articles [30, 36]. Most exercises reviewed were at least half a day in duration, with many designed to be delivered over one or two days [29–32, 34–36, 38]. Exercises that were shorter in duration involved less group discussion and networking [36] or were more task-oriented and designed to exercise specific tasks under constrained timelines (e.g., drafting a communications plan) [29, 31].

#### Exercise evaluation and improvement

##### Aligning evaluation methods with the purpose of the exercise

Aligning evaluation methods with the purpose of the exercise was an important insight emerging from the literature that critically examined how exercises were evaluated [42, 43]. For example, exercises conducted for accountability purposes typically involve making comparisons to show improvements over time or to compare to other jurisdictions or agencies. This requires high-level standardization of methods and measures [43]. It is also important to ensure there is a sufficient number of trained staff who can identify the root causes of the response failures observed during the exercise [42, 43]. When an agency’s preparedness will be assessed, it is recommended that both the agency’s capability to respond to the scenario, and the exercise design, be evaluated based on participant feedback [41, 43].

For exercises with a primary aim to enhance competencies, pre-post surveys have been found to be highly beneficial to assess the effectiveness of the exercise to meet learning objectives [29, 43]. These surveys can also be used to encourage participants to reflect on their own and their organization’s response capabilities [43]. This could include measures to assess what the participants learned and how it transferred to actual changes in their “on-the-job performance” via a follow-up survey [36]. The Kirkpatrick Model has been recommended to guide the development of pre-post survey items [43]. This model is commonly used to guide evaluations of training programs focusing on four levels of criteria: reaction, learning, behaviour, and results. In applying this model, an evaluation could focus on assessing perceived quality of the exercise, whether learning outcomes are achieved, what actions could be taken, and to also encourage participants to reflect on system capabilities [43].

Exercises that are focused on supporting improvement (i.e., identifying gaps or limitations in existing plans and processes) require evaluation data that are more detailed and tailored to the organization and context. The routine use of post-exercise debriefs (including hot debriefs and cold debriefs), has been recommended to support these types of developmental evaluations in addition to all other types of exercises and evaluations.

##### Facilitating ongoing improvement

How to embed activities following exercise completion to encourage an ongoing improvement approach was described in the literature including the use of debriefs as a strategy to bring participants or facilitators together for a discussion on issues or gaps identified and to gather feedback on the exercise itself [30, 32, 36, 38, 39, 41, 43]. One article specifically described the use of both hot debriefs and cold debriefs, where hot debriefs are conducted immediately after the exercise to capture instant reactions, and cold debriefs are carried out a few weeks later to allow participants a chance to reflect on the exercise [43]. An additional activity to encourage learning and transfer to practice included disseminating reports to participants following the exercise to summarize the results, including issues identified and opportunities for improvement [30, 41]. One article described an after-action conference where participating agencies discussed lessons learned and used a consensus-building process to develop a regional improvement plan [41]. The literature also described a few examples where participants were tasked with activities to help build action or improvement plans during the exercise [30, 34, 41, 43].

Table 3 summarizes the key insights on approaches to improve the design, conduct and evaluation of exercises for the public health agency context.

**Table 3:** Summary of research insights on how to optimize exercises for the public health agency context and proposed actions

Topic	Insights from the review:	Proposed action points
Establishing a Primary Aim for the Exercise	Better define the exercise aim and use this aim to direct exercise design choices (e.g., type of exercise, scenario design, discussion questions or tasks, level of facilitator involvement and evaluation methods).	Define the exercise aim early and revisit it throughout the planning process to ensure all aspects of the exercise design align.
Directing Exercise Content	Draw on various inputs to improve scenario design and enhance the discussion questions and activities.	Draw on various types of inputs to direct the focus of the exercise. Consider using after-action reports, core competencies, and information from current/past events.
Embedding Educational Components	Integrate educational components to enhance learning outcomes and support participants in identifying opportunities to improve policies and procedures.	Explore the value and feasibility of adding educational components to enhance learning outcomes.
Developing Realistic Scenarios	Focus on crafting detailed and plausible scenarios with a high degree of realism and psychological fidelity.	Apply various techniques to develop a realistic scenario. Consider tailoring exercises to national, regional or local contexts, using actual names of organizations, using details from real events, mirroring a realistic time frame, and incorporating some ambiguity in presenting information about the scenario early on.
Supporting Exercise Collaboration	Bring in a mix of agencies and roles to participate in the exercise and consider how to support lateral and vertical discussions. Consider power dynamics in planning how to facilitate exercise discussions.	Include facilitation techniques to support discussions between agencies and across different roles in the system. Reflect on how power dynamics may impact engagement and what adjustments can be made to support participation.
Reviewing Participant Representation	Often participant representation is identified as an area for improvement.	Review exercise plans considering what types of participants are needed to support exercise discussions/tasks. Provide a supporting rationale for including different roles and organizations. Map decisions involved in the exercise to roles needed to guide decision-making tasks.
Selecting and Supporting Exercise Facilitators	It is important to consider facilitator expertise (e.g., skills in facilitating small group discussion, content knowledge in the area and context) and invest time and resources to train facilitators well before the exercise.	Ensure exercise facilitators have strong facilitation skills, knowledge of the content area, and training before the exercise.
Focusing Exercise Discussions	Develop more specific discussion questions that will guide targeted and time-bound decision-making and ensure these questions align with participant roles and responsibilities. Exercise discussions need adequate time to support group discussion and networking.	Ensure exercise discussion questions and activities align with participant roles, include targeted and time-bound decisions and consider reducing the number of questions/activities to provide enough time for discussion and networking.
Aligning Evaluation Methods with the Purpose of the Exercise	The methods used in exercise evaluations need to align more closely with the primary aim of the exercise (e.g., methods for exercises aiming to assess organizational performance will be different from methods used for exercises that aim to enhance workforce competencies).	Use the primary aim of the exercise to direct the design of exercise evaluation methods and other learning activities. Draw on available evaluation frameworks to help select relevant questions and methods.
Facilitating Ongoing Improvement	The value of emergency preparedness exercises could be enhanced by investing in post-exercise activities (e.g., debriefs) to reflect on the exercise results and identify strengths and next steps for improvement.	Carry out post-exercise activities to reflect on the results involving people that can implement opportunities for improvement.

## Discussion

Emergency preparedness exercises are an integral component of emergency management programs that can support feedback and co-evolution amongst individuals and organizations to strengthen preparedness. As public health risks become more frequent and complex, optimizing the use of exercises in the public health agency context is critical, considering public health’s essential role in health system emergency management. This rapid review responded to a gap in the literature by synthesizing available information on core principles and practices that can be useful to enhance exercise outcomes, with a specific focus on exercises relevant to the public health agency context.

The literature reviewed highlights a range of practices relevant to exercise planning, implementation, and evaluation that can be used to strengthen the expected outcomes of emergency preparedness exercises. This included a focus on how scenarios are developed, how participants are selected and organized during an exercise, the importance of thoughtful selection and training of exercise facilitators, selecting evaluation methods that closely align with the purpose of the exercise, and the importance of embedding activities that will encourage pathways to improvement.

Similar to observations in other recent reviews on emergency preparedness exercises [11, 14], publications on public health emergency preparedness exercises stalled during the COVID-19 pandemic. There were also limited publications that met the eligibility criteria for this review that have evaluated the virtual delivery of exercises or that used other innovative technologies. However, these types of innovations are starting to be described in the literature, and it is anticipated that this will become an emerging area in exercise design [45–47]. The majority of included articles focused on tabletop exercises. This could be due to the unique value of tabletop exercises for the public health agency context or the more significant resources required to conduct operations-based exercises. It is important to keep this in mind when considering how to apply the key insights from this review to develop future exercises that take different forms. It was also noted that most of the included articles focused on exercises developed to train personnel or identify areas of a plan or response that need improvement. There were fewer exercises conducted for accountability purposes (e.g., demonstrating compliance with standards) with structured performance indicators. One exception was an article describing a series of functional exercises designed to test decision-making capacity among command centers in rural communities [41]. There is an opportunity to use public health-specific emergency preparedness frameworks and indicators to help direct the focus of exercise programs where response capacities can be tested against defined indicators [5, 48].

The critical appraisal of included studies highlighted that the quality of supporting evidence could be strengthened through more rigorous evaluation and identified opportunities to strengthen the reporting of evaluation methods. To enhance the quality of evaluation studies focusing on emergency preparedness exercises, there is a need for several advancements. First, studies should provide a transparent and detailed description of their evaluation methods, particularly their analytical approaches. Additionally, there should be a move towards the standardization of evaluation methods, with an increase use of evidence-based frameworks and consistent measures [5, 49]. Such standardization would improve comparability across different studies and exercises. This review identified a number of studies focused on best practices in exercise evaluation that could be applied to further strengthen and standardize evaluation approaches [42, 43, 49].

Building resilience in the public health system is essential to effectively respond to public health emergencies. When exercises are designed and delivered with quality using evidence-informed approaches, they can be used to strengthen other essential response capacities. For example, a key component in the Resilience Framework for Public Health Emergency Preparedness identifies how practice and experience, gained through emergency preparedness exercises or experiences in actual events or emergencies, can enable and reinforce other vital elements [5]. Ongoing evaluation of response plans and procedures can assist public health agencies in understanding if they are ready to respond in the event of an emergency, build relationships and collaborative networks, identify gaps and help guide opportunities for improvement [5]. There are also opportunities to utilize just in time training and simulation exercises to strengthen readiness for more high-priority and imminent threats such (e.g., detection of an emerging infectious disease in a neighbouring region with the potential to escalate in severity and scale) [50].

### Limitations

Rapid reviews are known to have some limitations [51]. The narrow research question did not support a comprehensive description of the types of exercises being conducted in the public health agency context. Other limitations included using a limited number of sources and restrictions on the languages of included studies and the dates searched. A systematic review is a more rigorous approach to knowledge synthesis and; for example, more extensive searching (across more databases and with fewer limits and restrictions) may have yielded additional information to expand on the themes in this study. Given the practical value of this research question to public health practitioners, the summary of evidence-based practices provided more quickly through a rapid review was important. A scoping review methodology is another option for knowledge synthesis and qualitative analysis approaches described for scoping reviews may have enhanced the description of the available literature on exercises conducted in the public health agency context. The high-level practical list of relevant strategies was deemed sufficient for the intended operational use of the rapid review findings.

Of the 15 articles included in this study, eight originated from the 2017 scoping review by Skryabina et al. that summarized literature published between 1990 and September 2015 [11]. That scoping review had a different focus, aiming to document the various types of outcomes of emergency exercises for personnel involved and for organizations. The advantage of screening all articles from this scoping review was to reanalyze this literature with a focus on more upstream considerations including factors that impacted the outcomes of the exercises under study. This step greatly expanded the information available for thematic analysis. In contrast, the search of grey literature did not yield any sources that met our inclusion criteria; however, that process was valuable for a secondary purpose related to this work, which was to identify existing practical resources and tools already available and consider opportunities for improvement.

The authors conducted a quality appraisal to identify gaps in reporting among the included studies; however, no papers were excluded from the final synthesis. This could have impacted the strength of some of the key insights from this review. Strengths of this review included its focus on gathering practical insights to guide emergency preparedness exercises, developing search strategies in collaboration with library information specialists, and involving multiple reviewers in study selection and data extraction. While this review has identified several areas for improvement based on key informant interviews and evaluation studies, there may be additional opportunities to enhance emergency preparedness exercises that haven’t been captured in this literature. One important aspect is being more intentional about when exercises are conducted. For instance, exercises could be prompted when gaps are identified after an emergency response, as part of capacity-building efforts, as an annual requirement, or in response to changes to policies or plans [10]. Further validation and expansion of the key insights from this review through expert consultation methods could be an interesting area of future research to temper the study’s limitations and continue to advance knowledge of evidence-informed practices around the design, conduct and evaluation of emergency preparedness exercises.

## Conclusion

This rapid review highlights insights relevant to the public health agency context on how to strengthen exercises with a focus on how scenarios are developed, how participants are selected and organized during an exercise, thoughtful selection and training of exercise facilitators, selecting evaluation methods that closely align with the purpose of the exercise, and the importance of embedding activities that will encourage pathways to improvement. Findings from this review can be utilized in practice to continue the important work of designing emergency preparedness exercises that strengthen the resilience of public health systems. There is a need for further studies on exercises conducted within the public health agency context, as well as research addressing current gaps in knowledge regarding evidence-informed practices in this area. There is a need for more guidance on triggers for exercises (such as risk assessments, recommendations from after-action reviews), additional research on innovative technologies and approaches to enhance participant engagement, and how to incorporate evidence-informed frameworks and indicators to strengthen exercise design and evaluation.

## Electronic supplementary material

Below is the link to the electronic supplementary material.

Supplementary Material 1

Supplementary Material 2

Supplementary Material 3

## Data Availability

The data generated and analyzed during this rapid review are available from the corresponding author on reasonable request.

## Abbreviations

COVID-19: Coronavirus Disease 2019
CASP: Critical Appraisal Skills Programme
OECD: Organisation for Economic Co-Operation and Development countries

## References

[1] Daniels RS, McCauley JW. The health of influenza surveillance and pandemic preparedness in the wake of the COVID-19 pandemic. J Gen Virol. 2023;104(2):001822. 10.1099/jgv.0.001822. 10.1099/jgv.0.001822 36800222

[2] Hertelendy AJ, Howard C, Sorensen C, Ranse J, Eboreime E, Henderson S, et al. Seasons of smoke and fire: Preparing health systems for improved performance before, during, and after wildfires. Lancet Planet Health. 2024;8(8):e588–602. 10.1016/S2542-5196(24)00144-X. 39122327 10.1016/S2542-5196(24)00144-X

[3] Polgreen PM, Polgreen EL. Emerging and re-emerging pathogens and diseases, and health consequences of a changing climate. Infect Dis. 2016;40–48:e2.

[4] Semenza JC. Lateral public health: advancing systemic resilience to climate change. Lancet Reg Health Eur. 2021;9:100231. 10.1016/j.lanepe.2021.100231. 34642677 10.1016/j.lanepe.2021.100231 PMC8495299

[5] Khan Y, O’Sullivan T, Brown A, Tracey S, Gibson J, Généreux M, et al. Public health emergency preparedness: A framework to promote resilience. BMC Public Health. 2018;18(1):1344. 10.1186/s12889-018-6250-7. 30518348 10.1186/s12889-018-6250-7 PMC6280369

[6] Belfroid E, Roβkamp D, Fraser G, Swaan C, Timen A. Towards defining core principles of public health emergency preparedness: scoping review and Delphi consultation among European union country experts. BMC Public Health. 2020;20(1):1482. 10.1186/s12889-020-09307-y. 32998729 10.1186/s12889-020-09307-y PMC7527265

[7] United States Department of Homeland Security. Homeland security exercise and evaluation program (HSEEP) [Internet]. Homeland Security; 2020 [cited 2025 Mar 25]. Available from: https://www.fema.gov/sites/default/files/2020-04/Homeland-Security-Exercise-and-Evaluation-Program-Doctrine-2020-Revision-2-2-25.pdf

[8] European Centre for Disease Prevention and Control. Health emergency preparedness for imported cases of high-consequence infectious diseases [Internet]. Stockholm: ECDC. 2019 [cited 2025 Mar 25]. Available from: https://www.ecdc.europa.eu/en/publications-data/health-emergency-preparedness-imported-cases-high-consequence-infectious-diseases

[9] International Health Regulations (IHR). HR monitoring and evaluation framework [Internet]. Geneva: World Health Organization; 2018. [cited 2025 Mar 25]. Available from: WHO-WHE-CPI-2018.51-eng.pdf.

[10] World Health Organization. WHO simulation exercise manual [Internet]. Geneva: World Health Organization. 2017 [cited 2025 Mar 25]. Available from: https://www.who.int/publications/i/item/WHO-WHE-CPI-2017.10

[11] Skryabina E, Reedy G, Amlot R, Jaye P, Riley P. What is the value of health emergency preparedness exercises? A scoping review study. Int J Disaster Risk Reduct. 2017;21:274–83. 10.1016/j.ijdrr.2016.12.010.

[12] Waring S, Moisi I, Barrett C, Gordts S. Identifying what components of full-scale emergency exercises improve disaster response learning: A rapid evidence assessment. Int J Disaster Risk Reduct. 2024;104:104390. 10.1016/j.ijdrr.2024.104390.

[13] Pek JH, Quah LJJ, Valente M, Ragazzoni L, Della Corte F. Use of simulation in full-scale exercises for response to disasters and mass-casualty incidents: a scoping review. Prehosp Disaster Med. 2023;38(6):792–806. 10.1017/S1049023X2300660X. 37997445 10.1017/S1049023X2300660X

[14] Mahdi SS, Jafri HA, Allana R, Battineni G, Khawaja M, Sakina S, et al. Systematic review on the current state of disaster Preparation simulation exercises (SimEx). BMC Emerg Med. 2023;23(1):52. 10.1186/s12873-023-00824-8. 37226121 10.1186/s12873-023-00824-8 PMC10206538

[15] Skryabina EA, Betts N, Reedy G, Riley P, Amlôt R. The role of emergency preparedness exercises in the response to a mass casualty terrorist incident: A mixed methods study. Int J Disaster Risk Reduct. 2020;46:101503. 10.1016/j.ijdrr.2020.101503. 33312855 10.1016/j.ijdrr.2020.101503 PMC7709486

[16] Verni C. A hospital system’s response to a hurricane offers lessons, including the need for mandatory interfacility drills. Health Aff. 2012;31(8):1814–21. 10.1377/hlthaff.2012.0154. 10.1377/hlthaff.2012.0154 22869660

[17] Agboola F, McCarthy T, Biddinger PD. Impact of emergency preparedness exercise on performance. J Public Health Manag Pract. 2013 Sep-Oct;19(Supplement 2):S77–83. 10.1097/PHH.0b013e31828ecd84. 10.1097/PHH.0b013e31828ecd84 23903400

[18] Fowkes V, Blossom HJ, Sandrock C, Mitchell B, Brandstein K. Exercises in emergency preparedness for health professionals in community clinics. J Community Health. 2010;35(5):512–8. 10.1007/s10900-010-9221-1. 20146093 10.1007/s10900-010-9221-1 PMC2952103

[19] Hamel C, Michaud A, Thuku M, Skidmore B, Stevens A, Nussbaumer-Streit B, et al. Defining rapid reviews: a systematic scoping review and thematic analysis of definitions and defining characteristics of rapid reviews. J Clin Epidemiol. 2021;129:74–85. 10.1016/j.jclinepi.2020.09.041. 33038541 10.1016/j.jclinepi.2020.09.041

[20] Tricco AC, Lillie E, Zarin W, O’Brien KK, Colquhoun H, Levac D, et al. PRISMA extension for scoping reviews (PRISMA-ScR): checklist and explanation. Ann Intern Med. 2018;169(7):467–73. 10.7326/M18-0850. 30178033 10.7326/M18-0850

[21] Ayiku L, Hudson T, Williams C, Levay P, Jacob C. The NICE OECD countries’ geographic search filters: part 2—validation of the MEDLINE and embase (Ovid) filters. J Med Libr Assoc. 2021;109(4). https://jmla.pitt.edu/ojs/jmla/article/view/1224 10.5195/jmla.2021.1224 PMC8608218 34858087

[22] Organization for Economic Co-operation and Development (OECD). Members and Partners [Internet]. Organization for Economic Co-operation and Development (OECD). 2024 [cited 2025 Mar 25]. Available from: https://www.oecd.org/about/members-and-partners/

[23] Veritas Health Innovation. Covidence systematic review software [Internet]. 2024 [cited 2025 Mar 25]. Available from: https://www.covidence.org

[24] CASP. CASP Qualitative Checklist [Internet]. Critical Appraisal Skills Programme. [cited 2025 Mar 25]. Available from: https://casp-uk.net/casp-tools-checklists/

[25] Zulu JM, Kinsman J, Michelo C, Hurtig AK. Integrating National community-based health worker programmes into health systems: a systematic review identifying lessons learned from low-and middle-income countries. BMC Public Health. 2014;14(1):987. 10.1186/1471-2458-14-987. 25245825 10.1186/1471-2458-14-987 PMC4192351

[26] King L, Harrington A, Linedale E, Tanner E. A mixed methods thematic review: Health-related decision‐making by the older person. J Clin Nurs. 2018;27(7–8):e1327–43. 10.1111/jocn.14261. 29322576 10.1111/jocn.14261

[27] CASP. CASP Systematic Review Checklist [Internet]. Critical Appraisal Skills Programme. [cited 2025 Mar 25]. Available from: https://casp-uk.net/casp-tools-checklists/

[28] Thomas J, Harden A. Methods for the thematic synthesis of qualitative research in systematic reviews. BMC Med Res Methodol. 2008;8(1):45. 10.1186/1471-2288-8-45. 18616818 10.1186/1471-2288-8-45 PMC2478656

[29] Freimuth VS, Hilyard KM, Barge JK, Sokler LA, Action. Not talk: A simulation of risk communication during the first hours of a pandemic. Health Promot Pract. 2008;9(4):S35–44. 10.1177/1524839908322111. 10.1177/1524839908322111 18936258

[30] High EH, Lovelace KA, Gansneder BM, Strack RW, Callahan B, Benson P. Promoting community preparedness: lessons learned from the implementation of a chemical disaster tabletop exercise. Health Promot Pract. 2010;11(3):310–9. 10.1177/1524839908325063. 19116424 10.1177/1524839908325063

[31] Macario E, Benton LD, Yuen J, Torres M, Macias-Reynolds V, Holsclaw P, et al. Preparing public health nurses for pandemic influenza through distance learning. Health Promot Pract. 2007;24(1):66–72. 10.1111/j.1525-1446.2006.00609.x. 10.1111/j.1525-1446.2006.00609.x 17214655

[32] Macario E, Heyden L, Nakahara N, Macias-Reynolds V. Preparing for pandemic influenza: California confronts the legal implications. Health Promot Pract. 2009;10(4):573–8. 10.1177/1524839907308118. 18434565 10.1177/1524839907308118

[33] Manageiro V, Caria A, Furtado C, Botelho A, Oleastro M, Gonçalves SC, et al. Intersectoral collaboration in a one health approach: lessons learned from a country-level simulation exercise. One Health. 2023;17:100649. 10.1016/j.onehlt.2023.100649. 38116455 10.1016/j.onehlt.2023.100649 PMC10728331

[34] Morris JG, Greenspan A, Howell K, Gargano LM, Mitchell J, Jones JL, et al. Southeastern center for emerging biologic threats tabletop exercise: foodborne toxoplasmosis outbreak on college campuses. Biosecur Bioterror. 2012;10(1):89–97. 10.1089/bsp.2011.0040. 22283568 10.1089/bsp.2011.0040 PMC3316480

[35] Sandström BE, Eriksson H, Norlander L, Thorstensson M, Cassel G. Training of public health personnel in handling CBRN emergencies: A table-top exercise card concept. Environ Int. 2014;72:164–9. 10.1016/j.envint.2014.03.009. 24742601 10.1016/j.envint.2014.03.009

[36] Sarpy SA, Warren CR, Kaplan S, Bradley J, Howe R. Simulating public health response to a severe acute respiratory syndrome (SARS) event: A comprehensive and systematic approach to designing, implementing, and evaluating a tabletop exercise. J Public Health Manag Pract. 2005;11(Supplement):S75–82. 10.1097/00124784-200511001-00013. 10.1097/00124784-200511001-00013 16205548

[37] Savoia E, Biddinger PD, Fox P, Levin DE, Stone L, Stoto MA. Impact of tabletop exercises on participants’ knowledge of and confidence in legal authorities for infectious disease emergencies. Disaster Med Public Health Prep. 2009;3(2):104–10. 10.1097/DMP.0b013e3181a539bc. 19491605 10.1097/DMP.0b013e3181a539bc

[38] So M, Dziuban EJ, Franks JL, Cobham-Owens K, Schonfeld DJ, Gardner AH, et al. Extending the reach of pediatric emergency preparedness: A virtual tabletop exercise targeting children’s needs. Public Health Rep. 2019;134(4):344–53. 10.1177/0033354919849880. 31095469 10.1177/0033354919849880 PMC6598136

[39] Dausey DJ, Buehler JW, Lurie N. Designing and conducting tabletop exercises to assess public health preparedness for manmade and naturally occurring biological threats. BMC Public Health. 2007;7(1):92. 10.1186/1471-2458-7-92. 17535426 10.1186/1471-2458-7-92 PMC1894789

[40] Ghiga I, Richardson S, Álvarez AMR, Kato M, Naidoo D, Otsu S, et al. PIPDeploy: development and implementation of a gamified table top simulation exercise to strengthen National pandemic vaccine preparedness and readiness. Vaccine. 2021;39(2):364–71. 10.1016/j.vaccine.2020.11.047. 33293160 10.1016/j.vaccine.2020.11.047 PMC7805265

[41] Obaid JM, Bailey G, Wheeler H, Meyers L, Medcalf SJ, Hansen KF, et al. Utilization of functional exercises to build regional emergency preparedness among rural health organizations in the US. Prehosp Disaster Med. 2017;32(2):224–30. 10.1017/S1049023X16001527. 28134064 10.1017/S1049023X16001527

[42] Savoia E, Agboola F, Biddinger P. A conceptual framework to measure systems’ performance during emergency preparedness exercises. Int J Environ Res Public Health. 2014;11(9):9712–22. 10.3390/ijerph110909712. 25233015 10.3390/ijerph110909712 PMC4199045

[43] Skryabina PDE, Riley PDP, Reedy PDG, Amlôt PDR. A scoping review of evaluation methods for health emergency preparedness exercises. Am J Disaster Med. 2018;13(2):107–27. 10.5055/ajdm.2018.0292. 30234917 10.5055/ajdm.2018.0292

[44] Straus SG, Lewis MW, Connor K, Eden R, Boyer ME, Marler T et al. Collective simulation-based training in the U.S. army: user interface fidelity, costs, and training effectiveness [Internet]. Santa Monica, CA: RAND Corporation. 2019. Available from: https://www.rand.org/pubs/research_reports/RR2250.html

[45] Luo Y, Li M, Tang J, Ren J, Zheng Y, Yu X, et al. Design of a virtual reality interactive training system for public health emergency preparedness for major emerging infectious diseases: theory and framework. JMIR Serious Games. 2021;9(4):e29956. 10.2196/29956. 34904951 10.2196/29956 PMC8715362

[46] Muhsen K, Cohen D, Glatman-Freedman A, Husseini S, Perlman S, McNeil C. Review of Israel’s action and response during the COVID-19 pandemic and tabletop exercise for the evaluation of readiness and resilience—lessons learned 2020–2021. Front Public Health. 2024;11:1308267. 10.3389/fpubh.2023.1308267. 38328537 10.3389/fpubh.2023.1308267 PMC10847317

[47] Alshowair A, Bail J, AlSuwailem F, Mostafa A, Abdel-Azeem A. Use of virtual reality exercises in disaster preparedness training: A scoping review. SAGE Open Med. 2024;12:20503121241241936. 10.1177/20503121241241936. 38623475 10.1177/20503121241241936 PMC11017811

[48] European Centre for Disease Prevention and Control. HEPSA: health emergency preparedness self-assessment tool: User guide [Internet]. ECDC. 2018. Available from: https://www.ecdc.europa.eu/sites/default/files/documents/Technical-Doc-HEPSA-tool-update-dec-18.pdf

[49] Khan Y, Brown AD, Gagliardi AR, O’Sullivan T, Lacarte S, Henry B, et al. Are we prepared? The development of performance indicators for public health emergency preparedness using a modified Delphi approach. PLoS ONE. 2019;14(12):e0226489. 10.1371/journal.pone.0226489. 31869359 10.1371/journal.pone.0226489 PMC6927653

[50] English R, Carlson H, Geduld H, Nyasulu JCY, Louw Q, Berner K, et al. Defining and identifying the critical elements of operational readiness for public health emergency events: a rapid scoping review. BMJ Glob Health. 2024;9(8):e014379. 10.1136/bmjgh-2023-014379. 39209763 10.1136/bmjgh-2023-014379 PMC11367384

[51] Munn Z, Pollock D, Barker TH, Stone J, Stern C, Aromataris E, et al. The dark side of rapid reviews: A retreat from systematic approaches and the need for clear expectations and reporting. Ann Intern Med. 2023;176(2):266–7. 10.7326/M22-2603. 36571838 10.7326/M22-2603

