# Supplementary material for: Enhancing the design, conduct and evaluation of public health emergency preparedness exercises: a rapid review
Source: BMC Public Health. 2025 Jul 3;25:2366. doi: 10.1186/s12889-025-23270-6 (PMC12224861; doi:10.1186/s12889-025-23270-6)
Supplement: Supplementary file 2 — Supplementary Material 2 [file 12889_2025_23270_MOESM2_ESM.docx]

**Additional file 2: Indexed database and grey literature search queries**

**Table S1. MEDLINE search query**

| # | Searches | Results |
| --- | --- | --- |
| 1 | ((table* adj2 simulat*) or ((walk-through* or run-through* or operation* or play-based or game-based or situational) adj2 (exercise* or scenario* or simulation*)) or tabletop or "table top" or DART or TTX or VTTX).ab,ti,kw,kf. | 12550 |
| 2 | Civil Defense/ or Extreme Weather/ or Extreme Cold Weather/ or Extreme Hot Weather/ or Extreme Heat/ or Avalanches/ or Biohazard Release/ or Bioterrorism/ or Chemical Hazard Release/ or Cyclonic Storms/ or Disaster Medicine/ or Disaster Planning/ or Disaster Victims/ or Disasters/ or Disease Outbreaks/ or Earthquakes/ or (Emergencies/ not (exp Emergency Service, Hospital/ or Emergency Medical Services/ or exp Surgical Procedures, Operative/)) or Epidemics/ or Floods/ or Landslides/ or Mass Casualty Incidents/ or Pandemics/ or Radioactive Hazard Release/ or Terrorism/ or Tidal Waves/ or Tornadoes/ or Tsunamis/ or Volcanic Eruptions/ or Wildfires/ | 307848 |
| 3 | ("extreme weather" or "extreme heat" or "hot weather" or "extreme cold" or heatdome* or "heat dome*" or "cold weather" or "cold snap*" or "overnight low*" or "polar vortex" or coldwave* or "cold wave*" or heatwave or "heat wave*" or "heat extremes" or windchill* or "wind chill*" or humid* or "heat island*" or "all hazard" or "all hazards" or "critical event*" or "critical incident*" or "forest fire*" or "mass casualt*" or "tidal wave*" or "wild fire*" or "wild land fire*" or "wildland fire*" or ((biohazard* or chemical* or radioactiv* or hazard*) adj3 (release* or spill* or accident*)) or avalanche* or bioterroris* or blizzard* or brushfire* or "brush fire*" or bushfire* or "bush fire*" or cyclone or cyclones or Chernobyl or disaster* or earthquake* or "earth quake*" or ((emergency or emergencies) not ((emergency or emergencies) adj2 (accident or care or service* or department* or room or rooms or unit or units or ward or wards or hospital* or medical* or surgery or surgeries or surgical*))) or epidemic* or Fukushima or ("health system" adj3 (overload* or overwhelm* or shock*)) or "public health preparedness" or flood* or hurricane* or landslide* or (outbreak* adj5 (COVID* or SARS* or "severe acute respiratory syndrome" or ebola* or H1N1* or zika or MERS or "middle east respiratory syndrome")) or storm* or terroris* or tornado* or tsunami* or typhoon* or volcano* or wildfire* or pandemic* or "public health emergency" or "public health emergencies" or "emergency management" or "emergency response" or "emergency preparedness" or "emergency planning").ab,kf,kf,ti. | 683173 |
| 4 | 2 or 3 | 798864 |
| 5 | "Centers for Disease Control and Prevention, U.S."/ or Community Health Nursing/ or Community Health Planning/ or Community Health Services/ or Community Integration/ or Community Medicine/ or Community Networks/ or Community Participation/ or Community-Based Participatory Research/ or Community-Institutional Relations/ or Health Education/ or Health Promotion/ or International Health Regulations/ or National Health Programs/ or Population Characteristics/ or Population Dynamics/ or *Population Groups/ or Population Health Management/ or Population Health/ or Public Health Administration/ or Public Health Nursing/ or Public Health Practice/ or Public Health Systems Research/ or Public Health/ or Regional Health Planning/ or Social Medicine/ or Sociology, Medical/ or State Health Plans/ or United States Public Health Service/ or Computer Simulation/ or Primary Prevention/og or Health Systems Plans/ or Forecasting/ or International Cooperation/ | 785080 |
| 6 | (((community or communities or county or counties or parish* or neighborhood* or neighbourhood* or municipal* or urban or rural or suburban or population) adj2 health) or "health authorit*" or "health department*" or "health education" or "health promotion" or "health protection" or "health system*" or "health unit*" or "public health" or ((region or regions or regional*) adj3 health) or PHU or PHUs or WHO or CDC or ECDC or PHAC or PHO or BCCDC or INSPQ or PAHO or "world health organi#ation" or "center* for disease control" or "centre* for disease control").ti,kw,kf. | 337281 |
| 7 | 5 or 6 | 1016541 |
| 8 | 1 and 4 and 7 | 96 |
| 9 | Evaluation Studies as Topic/ or Evaluation Study/ or Evaluation Study.pt. or *Retrospective Studies/ or Systems Analysis/ or Benchmarking/ or Quality Improvement/ or Quality Control/ or Total Quality Management/ or Efficiency, Organizational/ or Efficiency/ or Management Audit/ or Qualitative Research/ or "Task Performance and Analysis"/ or Program Evaluation/ or Delphi Technique/ or Needs Assessment/ or *"Surveys and Questionnaires"/ or Checklist/ or *Disaster Planning/st or Civil Defense/st or Public Health Administration/st or Public Health Practice/st or Public Health/st or Communicable Disease Control/st or Models, Organizational/ or Models, Theoretical/ or Implementation Science/ or *Research Design/ | 996806 |
| 10 | (evaluat* or example* or "lesson* learned" or "after-action review*" or "after action review*" or "in-action review*" or "in action review*" or implement* or initiativ* or model or apprais* or assess* or benchmark* or improv* or enhanc* or quality or ineffective* or efficac* or effective* or outcome* or audit* or analyz* or analys* or ((preparedness or response) adj3 (capacity or capacities or capability or capabilities)) or (quality adj3 (assess* or improv* or manag* or control* or assur*)) or models or toolkit* or took-kit* or schema* or pilot* or principles or program or programs or programme or programmes or roadmap* or "road map*" or strategy or strategies).ti,kw,kf. | 5623694 |
| 11 | (evaluat* or example* or "lesson* learned" or "after-action review*" or "after action review*" or "in-action review*" or "in action review*" or implement* or initiativ* or model or apprais* or assess* or benchmark* or improv* or enhanc* or quality or ineffective* or efficac* or effective* or outcome* or audit* or analyz* or analys* or ((preparedness or response) adj3 (capacity or capacities or capability or capabilities)) or (quality adj3 (assess* or improv* or manag* or control* or assur*)) or models or toolkit* or took-kit* or schema* or pilot* or principles or program or programs or programme or programmes or roadmap* or "road map*" or strategy or strategies).ab. not medline.st. | 3010003 |
| 12 | 9 or 10 or 11 | 8375585 |
| 13 | 1 and 7 and 12 | 180 |
| 14 | 1 and 4 and 12 | 254 |
| 15 | 8 or 13 or 14 | 422 |
| 16 | (Afghanistan/ or Argentina/ or Asia, Western/ or Asia/ or Bahrain/ or Bangladesh/ or Belize/ or Bhutan/ or Bolivia/ or Brazil/ or Central America/ or Developing Countries/ or Ecuador/ or El Salvador/ or exp Africa/ or exp Asia, Central/ or exp Asia, Northern/ or exp Asia, Southeastern/ or exp Caribbean Region/ or exp China/ or exp India/ or Far East/ or French Guiana/ or Guatemala/ or Guyana/ or Honduras/ or Iran/ or Iraq/ or Jordan/ or Kuwait/ or Latin America/ or Lebanon/ or Middle East/ or Mongolia/ or Nepal/ or Nicaragua/ or Oman/ or Pakistan/ or Panama/ or Paraguay/ or Peru/ or Qatar/ or Saudi Arabia/ or South America/ or Sri Lanka/ or Suriname/ or Syria/ or Taiwan/ or Turkey/ or United Arab Emirates/ or Uruguay/ or Venezuela/ or Yemen/) not (Austria/ or Baltic States/ or Belgium/ or Chile/ or Colombia/ or Costa Rica/ or Czech Republic/ or Developed Countries/ or Estonia/ or Europe/ or exp Australia/ or exp Canada/ or exp Denmark/ or exp France/ or exp Germany/ or exp Italy/ or exp Japan/ or exp Korea/ or exp Norway/ or exp United Kingdom/ or exp United States/ or Finland/ or Greece/ or Hungary/ or Iceland/ or Ireland/ or Israel/ or Latvia/ or Lithuania/ or Luxembourg/ or Mexico/ or Netherlands/ or New Zealand/ or North America/ or Poland/ or Portugal/ or Slovakia/ or Slovenia/ or Spain/ or Sweden/ or Switzerland/ or Turkey/) | 1230345 |
| 17 | 15 not 16 | 392 |
| 18 | limit 17 to last 10 years | 250 |
| 19 | limit 18 to english | 240 |
| 20 | remove duplicates from 19 | 240 |

**Table S2. EMBASE search query**

| # | Searches | Results |
| --- | --- | --- |
| 1 | ((table* adj2 simulat*) or ((walk-through* or run-through* or operation* or play-based or game-based or situational) adj2 (exercise* or scenario* or simulation*)) or tabletop or "table top" or DART or TTX or VTTX).ab,ti,kw,kf. | 14975 |
| 2 | emergency management/ or disaster management/ or disaster planning/ or disaster recovery/ or disaster resilience/ or disaster response/ or disaster mitigation/ or disaster preparedness/ or business continuity/ or emergency evacuation/ or civil defense/ or extreme weather/ or extreme cold weather/ or extreme hot weather/ or volcano/ or wildfire/ or forest fire/ or severe weather/ or heat wave/ or hurricane/ or tornado/ or avalanche/ or earthquake/ or storm surge/ or tsunami/ or disaster/ or humanitarian crisis/ or mass disaster/ or natural disaster/ or bomb/ or improvised explosive device/ or terrorism/ or bioterrorism/ or chemical terrorism/ | 77665 |
| 3 | ("extreme weather" or "extreme heat" or "hot weather" or "extreme cold" or heatdome* or "heat dome*" or "cold weather" or "cold snap*" or "overnight low*" or "polar vortex" or coldwave* or "cold wave*" or heatwave or "heat wave*" or "heat extremes" or windchill* or "wind chill*" or humid* or "heat island*" or "all hazard" or "all hazards" or "critical event*" or "critical incident*" or "forest fire*" or "mass casualt*" or "tidal wave*" or "wild fire*" or "wild land fire*" or "wildland fire*" or ((biohazard* or chemical* or radioactiv* or hazard*) adj3 (release* or spill* or accident*)) or avalanche* or bioterroris* or blizzard* or brushfire* or "brush fire*" or bushfire* or "bush fire*" or cyclone or cyclones or Chernobyl or disaster* or earthquake* or "earth quake*" or ((emergency or emergencies) not ((emergency or emergencies) adj2 (accident or care or service* or department* or room or rooms or unit or units or ward or wards or hospital* or medical* or surgery or surgeries or surgical*))) or epidemic* or Fukushima or ("health system" adj3 (overload* or overwhelm* or shock*)) or "public health preparedness" or flood* or hurricane* or landslide* or (outbreak* adj5 (COVID* or SARS* or "severe acute respiratory syndrome" or ebola* or H1N1* or zika or MERS or "middle east respiratory syndrome")) or storm* or terroris* or tornado* or tsunami* or typhoon* or volcano* or wildfire* or pandemic* or "public health emergency" or "public health emergencies" or "emergency management" or "emergency response" or "emergency preparedness" or "emergency planning").ab,kf,kf,ti. | 804044 |
| 4 | 2 or 3 | 827295 |
| 5 | social medicine/ or community medicine/ or preventive medicine/ or public health/ or public health problem/ or health education/ or health promotion/ or community care/ or community health nursing/ or preventive health service/ or public health service/ or community participation/ or citizen science/ or community program/ | 636995 |
| 6 | (((community or communities or county or counties or parish* or neighborhood* or neighbourhood* or municipal* or urban or rural or suburban or population) adj2 health) or "health authorit*" or "health department*" or "health education" or "health promotion" or "health protection" or "health system*" or "health unit*" or "public health" or ((region or regions or regional*) adj3 health) or PHU or PHUs or WHO or CDC or ECDC or PHAC or PHO or BCCDC or INSPQ or PAHO or "world health organi#ation" or "center* for disease control" or "centre* for disease control").ti,kw,kf. | 346759 |
| 7 | 5 or 6 | 867582 |
| 8 | 1 and 4 and 7 | 88 |
| 9 | "evaluation and follow up"/ or course evaluation/ or evaluation research/ or evaluation study/ or follow up/ or outcome assessment/ or quality control/ or benchmarking/ or total quality management/ or validation process/ or retrospective study/ or system analysis/ or organizational efficiency/ or program evaluation/ or program acceptability/ or program appropriateness/ or "program cost effectiveness"/ or program effectiveness/ or program efficacy/ or program feasibility/ or program impact/ or program sustainability/ or checklist/ or needs assessment/ or quality improvement study/ or productivity/ or *qualitative research/ or task performance/ or Delphi study/ | 4691513 |
| 10 | (evaluat* or example* or "lesson* learned" or "after-action review*" or "after action review*" or "in-action review*" or "in action review*" or implement* or initiativ* or model or apprais* or assess* or benchmark* or improv* or enhanc* or quality or ineffective* or efficac* or effective* or outcome* or audit* or analyz* or analys* or ((preparedness or response) adj3 (capacity or capacities or capability or capabilities)) or (quality adj3 (assess* or improv* or manag* or control* or assur*)) or models or toolkit* or took-kit* or schema* or pilot* or principles or program or programs or programme or programmes or roadmap* or "road map*" or strategy or strategies).ti,kw,kf. | 7291311 |
| 11 | (evaluat* or example* or "lesson* learned" or "after-action review*" or "after action review*" or "in-action review*" or "in action review*" or implement* or initiativ* or model or apprais* or assess* or benchmark* or improv* or enhanc* or quality or ineffective* or efficac* or effective* or outcome* or audit* or analyz* or analys* or ((preparedness or response) adj3 (capacity or capacities or capability or capabilities)) or (quality adj3 (assess* or improv* or manag* or control* or assur*)) or models or toolkit* or took-kit* or schema* or pilot* or principles or program or programs or programme or programmes or roadmap* or "road map*" or strategy or strategies).ab. not embase.st. | 9004655 |
| 12 | limit 11 to (cochrane library and (evidence based medicine or outcomes research)) | 271 |
| 13 | 9 or 10 or 11 | 15945080 |
| 14 | 1 and 4 and 13 | 439 |
| 15 | 1 and 7 and 13 | 140 |
| 16 | 8 or 12 or 14 or 15 | 800 |
| 17 | limit 16 to last 10 years | 556 |
| 18 | limit 17 to conference abstracts | 230 |
| 19 | 17 not 18 | 326 |
| 20 | limit 19 to embase status | 81 |
| 21 | limit 20 to last 10 years | 81 |
| 22 | limit 21 to english language | 76 |
| 23 | remove duplicates from 22 | 76 |

**Table S3. Global Health search query**

| # | Searches | Results |
| --- | --- | --- |
| 1 | ((table* adj2 simulat*) or ((walk-through* or run-through* or operation* or play-based or game-based or situational) adj2 (exercise* or scenario* or simulation*)) or tabletop or "table top" or DART or TTX or VTTX).ab,ti,id. | 1138 |
| 2 | ("extreme weather" or "extreme heat" or "hot weather" or "extreme cold" or heatdome* or "heat dome*" or "cold weather" or "cold snap*" or "overnight low*" or "polar vortex" or coldwave* or "cold wave*" or heatwave or "heat wave*" or "heat extremes" or windchill* or "wind chill*" or humid* or "heat island*" or "all hazard" or "all hazards" or "critical event*" or "critical incident*" or "forest fire*" or "mass casualt*" or "tidal wave*" or "wild fire*" or "wild land fire*" or "wildland fire*" or ((biohazard* or chemical* or radioactiv* or hazard*) adj3 (release* or spill* or accident*)) or avalanche* or bioterroris* or blizzard* or brushfire* or "brush fire*" or bushfire* or "bush fire*" or cyclone or cyclones or Chernobyl or disaster* or earthquake* or "earth quake*" or ((emergency or emergencies) not ((emergency or emergencies) adj2 (accident or care or service* or department* or room or rooms or unit or units or ward or wards or hospital* or medical* or surgery or surgeries or surgical*))) or epidemic* or Fukushima or ("health system" adj3 (overload* or overwhelm* or shock*)) or "public health preparedness" or flood* or hurricane* or landslide* or (outbreak* adj5 (COVID* or SARS* or "severe acute respiratory syndrome" or ebola* or H1N1* or zika or MERS or "middle east respiratory syndrome")) or storm* or terroris* or tornado* or tsunami* or typhoon* or volcano* or wildfire* or pandemic* or "public health emergency" or "public health emergencies" or "emergency management" or "emergency response" or "emergency preparedness" or "emergency planning").ab,ti,id. | 219157 |
| 3 | (((community or communities or county or counties or parish* or neighborhood* or neighbourhood* or municipal* or urban or rural or suburban or population) adj2 health) or "health authorit*" or "health department*" or "health education" or "health promotion" or "health protection" or "health system*" or "health unit*" or "public health" or ((region or regions or regional*) adj3 health) or PHU or PHUs or WHO or CDC or ECDC or PHAC or PHO or BCCDC or INSPQ or PAHO or "world health organi#ation" or "center* for disease control" or "centre* for disease control").ti,id. | 76625 |
| 4 | (evaluat* or example* or "lesson* learned" or "after-action review*" or "after action review*" or "in-action review*" or "in action review*" or implement* or initiativ* or model or apprais* or assess* or benchmark* or improv* or enhanc* or quality or ineffective* or efficac* or effective* or outcome* or audit* or analyz* or analys* or ((preparedness or response) adj3 (capacity or capacities or capability or capabilities)) or (quality adj3 (assess* or improv* or manag* or control* or assur*)) or models or toolkit* or took-kit* or schema* or pilot* or principles or program or programs or programme or programmes or roadmap* or "road map*" or strategy or strategies).ti,id. | 836622 |
| 5 | (evaluat* or example* or "lesson* learned" or "after-action review*" or "after action review*" or "in-action review*" or "in action review*" or implement* or initiativ* or model or apprais* or assess* or benchmark* or improv* or enhanc* or quality or ineffective* or efficac* or effective* or outcome* or audit* or analyz* or analys* or ((preparedness or response) adj3 (capacity or capacities or capability or capabilities)) or (quality adj3 (assess* or improv* or manag* or control* or assur*)) or models or toolkit* or took-kit* or schema* or pilot* or principles or program or programs or programme or programmes or roadmap* or "road map*" or strategy or strategies).ab. | 2988617 |
| 6 | 1 and 2 and 3 | 24 |
| 7 | 4 or 5 | 3080696 |
| 8 | 1 and 2 and 7 | 100 |
| 9 | 1 and 3 and 7 | 32 |
| 10 | 6 or 8 or 9 | 108 |
| 11 | limit 10 to yr="2013 -Current" | 73 |
| 12 | limit 11 to english language | 69 |
| 13 | remove duplicates from 12 | 69 |

**Table S4. Business Continuity & Disaster Recovery Reference Center search query**

| # | Query | Results |
| --- | --- | --- |
| S1 | TI ( ((table* N2 simulat*) or ((walk-through* or run-through* or operation* or play-based or game-based or situational) N2 (exercise* or scenario* or simulation*)) or tabletop or "table top" or DART or TTX or VTTX) ) OR AB ( ((table* N2 simulat*) or ((walk-through* or run-through* or operation* or play-based or game-based or situational) N2 (exercise* or scenario* or simulation*)) or tabletop or "table top" or DART or TTX or VTTX) ) OR KW ( ((table* N2 simulat*) or ((walk-through* or run-through* or operation* or play-based or game-based or situational) N2 (exercise* or scenario* or simulation*)) or tabletop or "table top" or DART or TTX or VTTX) ) | 244 |
| S2 | DE "EXTREME weather" OR DE "HEAT waves (Meteorology)") OR DE "INDIAN Ocean Tsunami, 2004" OR DE "NATURAL disasters" OR DE "DROUGHTS" OR DE "EARTHQUAKES" OR DE "FLOODS" OR DE "FOREST fires" OR DE "HURRICANES" OR DE "MUDSLIDES" OR DE "STORMS" OR DE "TORNADOES" OR DE "TSUNAMIS" OR DE "VOLCANIC eruptions" OR DE "AVALANCHES" OR DE "MASS casualties" OR DE "CIVIL defense" OR DE "TERRORISM" OR DE "BIOTERRORISM" OR DE "BOMBINGS" OR DE "CHEMICAL terrorism" OR DE "DOMESTIC terrorism" OR DE "CRIMES against public safety" OR DE "RIOTS" OR DE "PUBLIC safety" | 19,420 |
| S3 | TI ( ("extreme weather" or "extreme heat" or "hot weather" or "extreme cold" or heatdome* or "heat dome*" or "cold weather" or "cold snap*" or "overnight low*" or "polar vortex" or coldwave* or "cold wave*" or heatwave or "heat wave*" or "heat extremes" or windchill* or "wind chill*" or humid* or "heat island*" or "all hazard" or "all hazards" or "critical event*" or "critical incident*" or "forest fire*" or "mass casualt*" or "tidal wave*" or "wild fire*" or "wild land fire*" or "wildland fire*" or ((biohazard* or chemical* or radioactiv* or hazard*) N3 (release* or spill* or accident*)) or avalanche* or bioterroris* or blizzard* or brushfire* or "brush fire*" or bushfire* or "bush fire*" or cyclone or cyclones or Chernobyl or disaster* or earthquake* or "earth quake*" or ((emergency or emergencies) not ((emergency or emergencies) N2 (accident or care or service* or department* or room or rooms or unit or units or ward or wards or hospital* or medical* or surgery or surgeries or surgical*))) or epidemic* or Fukushima or ("health system" N3 (overload* or overwhelm* or shock*)) or "public health preparedness" or flood* or hurricane* or landslide* or (outbreak* N5 (COVID* or SARS* or "severe acute respiratory syndrome" or ebola* or H1N1* or zika or MERS or "middle east respiratory syndrome")) or storm* or terroris* or tornado* or tsunami* or typhoon* or volcano* or wildfire* or pandemic* or "public health emergency" or "public health emergencies" or "emergency management" or "emergency response" or "emergency preparedness" or "emergency planning") ) OR AB ( ("extreme weather" or "extreme heat" or "hot weather" or "extreme cold" or heatdome* or "heat dome*" or "cold weather" or "cold snap*" or "overnight low*" or "polar vortex" or coldwave* or "cold wave*" or heatwave or "heat wave*" or "heat extremes" or windchill* or "wind chill*" or humid* or "heat island*" or "all hazard" or "all hazards" or "critical event*" or "critical incident*" or "forest fire*" or "mass casualt*" or "tidal wave*" or "wild fire*" or "wild land fire*" or "wildland fire*" or ((biohazard* or chemical* or radioactiv* or hazard*) N3 (release* or spill* or accident*)) or avalanche* or bioterroris* or blizzard* or brushfire* or "brush fire*" or bushfire* or "bush fire*" or cyclone or cyclones or Chernobyl or disaster* or earthquake* or "earth quake*" or ((emergency or emergencies) not ((emergency or emergencies) N2 (accident or care or service* or department* or room or rooms or unit or units or ward or wards or hospital* or medical* or surgery or surgeries or surgical*))) or epidemic* or Fukushima or ("health system" N3 (overload* or overwhelm* or shock*)) or "public health preparedness" or flood* or hurricane* or landslide* or (outbreak* N5 (COVID* or SARS* or "severe acute respiratory syndrome" or ebola* or H1N1* or zika or MERS or "middle east respiratory syndrome")) or storm* or terroris* or tornado* or tsunami* or typhoon* or volcano* or wildfire* or pandemic* or "public health emergency" or "public health emergencies" or "emergency management" or "emergency response" or "emergency preparedness" or "emergency planning") ) OR KW ( ("extreme weather" or "extreme heat" or "hot weather" or "extreme cold" or heatdome* or "heat dome*" or "cold weather" or "cold snap*" or "overnight low*" or "polar vortex" or coldwave* or "cold wave*" or heatwave or "heat wave*" or "heat extremes" or windchill* or "wind chill*" or humid* or "heat island*" or "all hazard" or "all hazards" or "critical event*" or "critical incident*" or "forest fire*" or "mass casualt*" or "tidal wave*" or "wild fire*" or "wild land fire*" or "wildland fire*" or ((biohazard* or chemical* or radioactiv* or hazard*) N3 (release* or spill* or accident*)) or avalanche* or bioterroris* or blizzard* or brushfire* or "brush fire*" or bushfire* or "bush fire*" or cyclone or cyclones or Chernobyl or disaster* or earthquake* or "earth quake*" or ((emergency or emergencies) not ((emergency or emergencies) N2 (accident or care or service* or department* or room or rooms or unit or units or ward or wards or hospital* or medical* or surgery or surgeries or surgical*))) or epidemic* or Fukushima or ("health system" N3 (overload* or overwhelm* or shock*)) or "public health preparedness" or flood* or hurricane* or landslide* or (outbreak* N5 (COVID* or SARS* or "severe acute respiratory syndrome" or ebola* or H1N1* or zika or MERS or "middle east respiratory syndrome")) or storm* or terroris* or tornado* or tsunami* or typhoon* or volcano* or wildfire* or pandemic* or "public health emergency" or "public health emergencies" or "emergency management" or "emergency response" or "emergency preparedness" or "emergency planning") ) | 60,668 |
| S4 | S2 OR S3 | 62,725 |
| S5 | DE "PUBLIC health" OR DE "COMMUNITY health services" OR DE "ENVIRONMENTAL health" OR DE "HEALTH planning" OR DE "POPULATION health" OR DE "PUBLIC health communication" OR DE "RURAL health" OR DE "SOCIAL medicine" OR DE "URBAN health" OR DE "PREVENTIVE medicine" OR DE "PUBLIC health administration" OR DE "PUBLIC health advisory groups" OR DE "PUBLIC health research" | 2,568 |
| S6 | TI ( ((community or communities or county or counties or parish* or neighborhood* or neighbourhood* or municipal* or urban or rural or suburban or population) N2 health) or "health authorit*" or "health department*" or "health education" or "health promotion" or "health protection" or "health system*" or "health unit*" or "public health" or ((region or regions or regional*) N3 health) or PHU or PHUs or WHO or CDC or ECDC or PHAC or PHO or BCCDC or INSPQ or PAHO or "world health organi?ation" or "center* for disease control" or "centre* for disease control") ) OR KW ( ((community or communities or county or counties or parish* or neighborhood* or neighbourhood* or municipal* or urban or rural or suburban or population) N2 health) or "health authorit*" or "health department*" or "health education" or "health promotion" or "health protection" or "health system*" or "health unit*" or "public health" or ((region or regions or regional*) N3 health) or PHU or PHUs or WHO or CDC or ECDC or PHAC or PHO or BCCDC or INSPQ or PAHO or "world health organi?ation" or "center* for disease control" or "centre* for disease control") ) | 2,600 |
| S7 | S5 OR S6 | 4,672 |
| S8 | S1 AND S4 AND S7 | 6 |
| S9 | DE "EVALUATION" OR DE "EDUCATIONAL evaluation" OR DE "EVALUATION research (Social action programs)" OR DE "HEALTH impact assessment" OR DE "HEALTH outcome assessment" OR DE "OUTCOME assessment (Social services)" OR DE "RISK assessment" OR DE "EVALUATION research" OR DE "STANDARDS" | 7,185 |
| S10 | TI ( (evaluat* or example* or "lesson* learned" or "after-action review*" or "after action review*" or "in-action review*" or "in action review*" or implement* or initiativ* or model or apprais* or assess* or benchmark* or improv* or enhanc* or quality or ineffective* or efficac* or effective* or outcome* or audit* or analyz* or analys* or ((preparedness or response) N3 (capacity or capacities or capability or capabilities)) or (quality N3 (assess* or improv* or manag* or control* or assur*)) or models or toolkit* or took-kit* or schema* or pilot* or principles or program or programs or programme or programmes or roadmap* or "road map*" or strategy or strategies) ) OR KW ( (evaluat* or example* or "lesson* learned" or "after-action review*" or "after action review*" or "in-action review*" or "in action review*" or implement* or initiativ* or model or apprais* or assess* or benchmark* or improv* or enhanc* or quality or ineffective* or efficac* or effective* or outcome* or audit* or analyz* or analys* or ((preparedness or response) N3 (capacity or capacities or capability or capabilities)) or (quality N3 (assess* or improv* or manag* or control* or assur*)) or models or toolkit* or took-kit* or schema* or pilot* or principles or program or programs or programme or programmes or roadmap* or "road map*" or strategy or strategies) ) | 0 |
| S11 | S9 OR S10 | 54,365 |
| S12 | S1 AND S7 AND S11 | 2 |
| S13 | S1 AND S4 AND S11 | 46 |
| S14 | S8 OR S12 OR S13 | 50 |
| S15 | S14 Limiters - Publication Date: 20130101- | 26 |

**Grey literature search queries**

**Search strategies:**

emergency OR disaster OR pandemic OR outbreak preparedness OR simulation OR response "tabletop exercise" OR TTX "public health" OR "community health" guide OR guidance OR primer OR example OR toolkit

emergency OR disaster OR pandemic OR outbreak preparedness OR simulation OR response "tabletop exercise" OR TTX "public health" OR "community health" design OR designing OR condut OR conducting OR evaluating OR evaluation OR evaluate OR assess OR assessment

emergency OR disaster OR pandemic OR outbreak "role play" OR roleplay "tabletop exercise" OR TTX "public health" OR "community health" how-to OR "best practice" OR "ready-made" OR sample OR template OR instruction

Terrorism OR "mass casualty" OR terrorist OR bioterrorism preparedness OR simulation OR response "tabletop exercise" OR TTX "public health" OR "community health" guide OR guidance OR primer OR example OR toolkit

Terrorism OR "mass casualty" OR terrorist OR bioterrorism preparedness OR simulation OR response "tabletop exercise" OR TTX "public health" OR "community health" design OR designing OR condut OR conducting OR evaluating OR evaluation OR evaluate OR assess OR assessment

Terrorism OR "mass casualty" OR terrorist OR bioterrorism "role play" OR roleplay "tabletop exercise" OR TTX "public health" OR "community health" how-to OR "best practice" OR "ready-made" OR sample OR template OR instruction

**Grey literature databases searched:**

Custom search engines (n = 3)

[**Canadian Health Departments and Agencies**](https://cse.google.com/cse?cx=54dae8807550ea08a)

[**US State Government Websites**](https://cse.google.com/cse?cx=f7f9d691627b6ed48)

[**International Public Health Resources**](https://cse.google.com/cse?cx=b15f5d17bb6cc614d)

Site specific searching (n = 7)

- Centers for Disease Control and Prevention: site:.cdc.gov
- European Centre for Disease Prevention and Control: site:.europa.eu
- Government of the United Kingdom: site:.gov.uk
- Government of New Zealand: site:.govt.nz
- Government of Australia: site:.gov.au
- Pan American Health Organization site:.paho.int/
- World Health Organization: site:.who.int/
