# Supplementary material for: Enhancing the design, conduct and evaluation of public health emergency preparedness exercises: a rapid review
Source: BMC Public Health. 2025 Jul 3;25:2366. doi: 10.1186/s12889-025-23270-6 (PMC12224861; doi:10.1186/s12889-025-23270-6)
Supplement: Supplementary file 3 — Supplementary Material 3 [file 12889_2025_23270_MOESM3_ESM.docx]

**Additional file 3: Additional information on the approach to critical appraisal and results**

Fourteen of the 15 included studies in this review were appraised with the Critical Appraisal Skills Program (CASP) checklist for qualitative studies.^1^ Results of this assessment are presented in Table S1. An overview of how items in this tool were adapted for relevance to this review is described below. Individual results can be made available upon request. Skryabina et al. (2008)^2^ was appraised with the CASP checklist for systematic reviews.^3^ Results of that assessment can be found at the end of this document.

**Table S1: Summary of results from the appraisal of qualitative and mixed-methods studies with a modified version of the CASP Checklist – Qualitative Studies^1^**

|  | Question 1 | Question 2 | Question 3 | Question 4 | Question 5 | Question 6 | Question 7 |
| --- | --- | --- | --- | --- | --- | --- | --- |
| Sarpy 2005 [4] | Yes | Yes | Yes | No | Yes | Yes | Yes |
| Dausey 2007 [5] | Yes | Yes | No | No | No | Unclear | Yes |
| Macario 2009 [6] | No | Unclear | No | No | No | No | No |
| Macario 2006 [7] | No | Unclear | No | No | Yes | No | No |
| Freimuth 2008 [8] | Yes | Yes | Yes | No | Yes | No | Yes |
| Morris 2012 [9] | No | Yes | Yes | No | No | No | No |
| Savoia 2014 [10] | No | Yes | Yes | Yes | Yes | Yes | Yes |
| Obaid 2017 [11] | No | Unclear | Yes | No | No | No | No |
| Manageiro 2023 [12] | No | Unclear | No | No | No | Unclear | No |
| Savoia 2009 [13] | Yes | Yes | Yes | No | Yes | Yes | Yes |
| High 2010 [14] | Yes | Yes | No | No | No | No | Yes |
| Sandrom 2014 [15] | No | Unclear | No | No | Yes | No | Yes |
| So 2019 [16] | Yes | Yes | Yes | Yes | Yes | Yes | Yes |
| Ghiga 2021 [17] | No | Unclear | No | No | No | No | No |

^1^Please note that the appraisal results for Skryabina et al. (2018) were not included in Table 1 as it required the CASP checklist for systematic reviews. Results are reported at the end of this document.

# **CASP – Qualitative Studies Checklist**

The seven questions used to appraise the studies listed in Table S1 are listed below along with notes on how the CASP items for qualitative studies were adapted.^1^

1. Are the research/evaluations questions or objectives clearly stated

- Adapted from CASP Q1
- If the paper includes research/evaluation questions or objectives

1. Was the approach (i.e., evaluation/research methodology) appropriate to address the aims of the study?

- Adapted from Q3 and Q2
- If the authors justify the design (e.g., have they discussed how they decided which method to use)
- If the methodology and study design (e.g., qualitative research, mixed methods evaluation) aligned with the study aims

1. Is the method of data collection clearly described?

- Adapted from CASP Q5
- If it is clear how data were collected
- If the researcher/authors has made the methods explicit (e.g., for interview method, is there an indication of how interview were conducted, or did they use a topic guide)

1. Is the role of the researcher clearly described?

- Adapted from CASP Q6
- If it is clear who analyzed the data and whether the authors are the exercise designers/facilitators/evaluators

1. Were the participants clearly described?

- Adapted from CASP 4
- If the number of participants contributing to the study are described
- If the role of participants in the exercise (i.e., players, facilitators, evaluators) are described

1. Was the data analysis sufficiently rigorous?

- CASP Q8
- If there is an in-depth description of the analysis process
- If sufficient data are presented to support the finding
- If lessons learned are supported by a comprehensive discussion

1. Is there a clear statement of findings?

- CASP Q9
- If findings are explicit
- If there is adequate discussion of the evidence both for and against the researcher’s / authors arguments
- If the researcher/author discussed the credibility of their findings (e.g., triangulation, respondent validation, more than one analyst)

# **CASP – Systematic Reviews**

Skryabina et al. (2018)^2^ was appraised with the CASP tool for systematic reviews.^3^

1. Did the review address a clearly focused question?

Yes No Unclear  Not applicable

Included in the method section on p 109. As this is a scoping review, they used a broader question that wasn’t defined with PICO. The research question that informed the scoping review study was: what is known from the existing literature about the effectiveness of public health emergency preparedness exercises and their evaluation methods? For this proportion of the scoping review on evaluation approaches, the scoping review could have benefited from some more specific sub questions to frame the focus of the review.

1. Did the authors look for the right type of papers?

Yes No Unclear  Not applicable

Inclusion criteria for this component of the scoping review are described in the methods with a rationale focusing on the aim to identify articles describing exercise evaluations where the methods are sufficiently described (p 109). The study designs (mostly evaluation studies) were appropriate for the aim of this scoping review.

1. Do you think all the important, relevant studies were included?

Yes No Unclear  Not applicable

Multiple electronic databases were searched including MEDLINE, CINAHL, EMBASE and Global Health. The search strategy was tailored to the scoping review methodology aiming to use more broad search terms in two areas (terms related to types of emergency situations and terms related to exercises) to identify articles on emergency preparedness exercises. The search strategy is described in the companion article. The use of Subject Headings and Indexing terms are described. The search strategy could have been supplement with other strategies including a search of grey literature, follow up from reference lists, personal contact with experts, etc. The original search strategy aimed to identify papers reporting on emergency exercise benefits without a specific purpose on exercise evaluation methods; this may have limited the available evidence related to emergency exercises’ evaluation discussed in this review. However, the authors were able to identify 64 studies for this analysis.

1. Did the review’s authors do enough to assess quality of the included studies?

Yes No Unclear  Not applicable

I’ve indicated yes, as this was a scoping review which follows a different process. In the review of evaluation methods as part of this scoping review, the authors are aiming to identify gaps and opportunities to improve studies in this area.

1. If the results of the review have been combined, was it reasonable to do so?

Yes No Unclear  Not applicable

The authors have catalogued the literature in this area to establish patterns in how exercises have been evaluated. A narrative synthesis follows.

1. What are the overall results of the review?

The review’s bottom line results could be described as: studies included in this review clearly demonstrated heterogeneity of evaluation methods utilized for evaluation of health emergency preparedness exercises. The authors found a lack of consistency, lack of justification, and lack of guidance in choosing an appropriate evaluation method and a fragmented and inconsistent approach to collecting data from exercise participants. The authors present a series of recommendations for improving the evaluation of different types of exercises.

1. How precise are the results?

Yes No Unclear  Not applicable

This review did not use quantitative methods for the synthesis of evidence.

1. Can the results be applied to the local population?

Yes No Unclear  Not applicable

While the majority of studies were conducted in the U.S., the recommendations on exercise evaluation are transferable and would be relevant to the public health agency context.

1. Were all important outcomes considered?

Yes No Unclear  Not applicable

The authors provided a comprehensive overview of evaluation methods used across studies.

1. Are the benefits worth the harms and costs?

Yes No Unclear  Not applicable

# **References**

1. Critical Appraisal Skills Programme. CAPS Qualitative Checklist [Internet]. Oxford: CASP UK; [cited 2024 Jun 24]. Available from: <https://casp-uk.net/casp-tools-checklists/>.
2. Skryabina E, Riley P, Reedy G, Amlot R. A scoping review of evaluation methods for health emergency preparedness exercises. Am J Disaster Med. 2018;13(2):107-127. <https://doi.org/10.5055/ajdm.2018.0292>.
3. Critical Appraisal Skills Programme. CAPS Systematic Review Checklist [Internet]. Oxford: CASP UK; [cited 2024 Jun 24]. Available from: <https://casp-uk.net/casp-tools-checklists/>.
4. Sarpy SA, Warren CR, Kaplan S, Bradley J, Howe R. Simulating public health response to a severe acute respiratory syndrome (SARS) event: a comprehensive and systematic approach to designing, implementing, and evaluating a tabletop exercise. J Public Health Manag Pract. 2005;Suppl:S75-82. <https://doi.org/10.1097/00124784-200511001-00013>.
5. Dausey DJ, Buehler JW, Lurie N. Designing and conducting tabletop exercises to assess public health preparedness for manmade and naturally occurring biological threats. BMC Public Health 2007;7:92. <https://doi.org/10.1186/1471-2458-7-92>.
6. Macario E, Benton LD, Yuen J, Torres M, Macias-Reynolds V, Holsclaw P, et al. Preparing public health nurses for pandemic influenza through distance learning. Public Health Nurs. 2007;24(1):66-72. <https://doi.org/10.1111/j.1525-1446.2006.00609.x>.
7. Macario E, Heyden L, Nakahara N, Macias-Reynolds V. Preparing for pandemic influenza: California confronts the legal implications. Health Promot Pract. 2009;10(4):573-578. <https://doi.org/10.1177/1524839907308118>.
8. Freimuth VS, Hilyard KM, Barge JK, Sokler LA. Action, not talk: a simulation of risk communication during the first hours of a pandemic. Health Promot Pract. 2008;9(4):35S-44S. <https://doi.org/10.1177/1524839908322111>.
9. Morris JG, Greenspan A, Howell K, Gargano LM, Mitchell J, Jones JL, et al. Southeastern Center for Emerging Biologic Threats tabletop exercise: foodborne toxoplasmosis outbreak on college campuses. Biosecur Bioterror. 2012;10(1):89-97. https://doi.org/[10.1089/bsp.2011.0040](https://doi.org/10.1089/bsp.2011.0040).
10. Savoia E, Biddinger PD, Fox P, Levin DE, Stone L, Stoto MA. Impact of tabletop exercises on participants' knowledge of and confidence in legal authorities for infectious disease emergencies. Disaster Med Public Health Prep. 2009;3(2):104-110. <https://doi.10.1097/DMP.0b013e3181a539bc>.
11. Obaid JM, Bailey G, Wheeler H, Meyers L, Medcalf SJ, Hansen KF, et al. Utilization of functional exercises to build regional emergency preparedness among rural health organizations in the US. Prehospital Disaster Med. 2017;32(2):224-230. <https://doi.org/10.1017/S1049023X16001527>.
12. Manageiro V, Caria A, Furtado C, Botelho A, Oleastro M, Goncalves SC. Intersectoral collaboration in a One Health approach: Lessons learned from a country-level simulation exercise. One Health. 2023;17. <https://doi.org/10.1016/j.onehlt.2023.100649>.
13. Savoia E, Agboola F, Biddinger PD. A conceptual framework to measure systems' performance during emergency preparedness exercises. Int J Environ Res Public Health. 2014;11(9):9712-9722. https://doi.[10.3390/ijerph110909712](https://doi.org/10.3390%2Fijerph110909712).
14. High EH, Lovelace KA, Gansneder BM, Strack RW, Callahan B, Benson P. Promoting community preparedness: lessons learned from the implementation of a chemical disaster tabletop exercise. Health Promot Pract 2010;11(3):310-319. https://doi.org/ 10.1177/1524839908325063.
15. Sandstrom BE, Eriksson H, Norlander L, Thorstensson M, Cassel G. Training of public health personnel in handling CBRN emergencies: a table-top exercise card concept. Environ Int. 2014;72:164-9. <https://doi.org/10.1016/j.envint.2014.03.009>.
16. So M, Dziuban EJ, Franks JL, Cobham-Owens K, Schonfeld DJ, Gardner AH, et al. Extending the reach of pediatric emergency preparedness: A virtual tabletop exercise targeting children's needs. Public Health Rep. 2019;134(4):344-353. <https://doi.org/10.1177/0033354919849880>.
17. Ghiga I, Richardson S, Alvarez AMR, Kato M, Naidoo D, Otsu S, et al. PIPDeploy: Development and implementation of a gamified table top simulation exercise to strengthen national pandemic vaccine preparedness and readiness. Vaccine. 2021;39(2):364-371. https://doi.org/ 10.1016/j.vaccine.2020.11.04.
